# Health-related quality of life and hypertension in people with HIV on long-term antiretroviral therapy in Uganda

**DOI:** 10.1371/journal.pone.0306928

**Published:** 2024-08-08

**Authors:** Charles Batte, Andrew Weil Semulimi, John Mukisa, Mariam Nakabuye, Jasper Nidoi, David Mukunya, Rosalind Parkes Ratanshi, Barbara Castelnuovo, Mohammed Lamorde, David Meya, William Checkley, Robert Kalyesubula, Trishul Siddharthan, Joseph B. Babigumira

**Affiliations:** 1 Department of Medicine, Lung Institute, School of Medicine, College of Health Sciences, Makerere University, Kampala, Uganda; 2 Department of Immunology and Molecular Biology, School of Biomedical Sciences, College of Health Sciences, Makerere University, Kampala, Uganda; 3 Department of Community and Public Health, Faculty of Health Sciences, Busitema University, Tororo, Uganda; 4 Infectious Diseases Institute, Makerere University, Kampala, Uganda; 5 Department of Psychiatry, University of Cambridge, Cambridge, United Kingdom; 6 Department of Medicine, School of Medicine, College of Health Sciences, Makerere University, Kampala, Uganda; 7 Center for Global Non-Communicable Diseases Research and Training, Department of Medicine, Johns Hopkins University, Baltimore, Maryland, United States of America; 8 Department of Physiology, School of Biomedical Sciences, College of Health Sciences, Makerere University, Kampala, Uganda; 9 Department of Medicine, Miller School of Medicine, University of Miami, Miami, Florida, United States of America; 10 Saw Swee Hock School of Public Health, National University of Singapore, Singapore, Singapore; Pan American Health Organization, UNITED STATES OF AMERICA

## Abstract

**Introduction:**

The presence of hypertension could reduce the health-related quality of life (HRQoL) of people with HIV (PWH). Yet, literature describing the HRQoL of PWH who have hypertension in Uganda is scarce making the design of locally adapted interventions cumbersome. In our study, we compared HRQoL scores of people with HIV with and without hypertension on long term antiretroviral therapy (ART) in Uganda.

**Methods:**

We recruited 149 PWH with hypertension and 159 PWH without hypertension in the long-term ART cohort at an urban clinic in Kampala, Uganda. Data on socio-demographics were collected using an interviewer designed questionnaire while data on the World Health Organisation clinical stage viral load and CD4 count as well as ART duration were extracted from clinic electronic database and a generic EuroQol -5D- 5L (EQ-5D- 5L) and Medical Outcome Study (MOS-HIV) questionnaire used to collect HRQoL data. Data were summarized using descriptive statistics while inferential statistics were used to determine associations between key variables and HRQoL. Mann-Whitney U tests were used to compare HRQoL between groups of interest.

**Results:**

One hundred ninety (61.7%) participants were female. PWH who had hypertension were older (Mean ± SD: 53.7 ± 8.3 vs 49.9 ± 8.6, p value <0.001) than those without hypertension. Participants with hypertension had lower overall median health utility scores (0.71 (0.33–0.80) vs 0.80 (0.44–0.80), p value = 0.029) and mean physical health score (48.44 ± 10.17 vs 51.44 ± 9.65, p value < 0.001) as opposed to those without hypertension. Hypertension (p value = 0.023), high income status, >70,000 UGX, (p value = 0.044), disclosure of the HIV status of the participants to their partner (p value = 0.026), and current history of smoking (p value = 0.029) were associated with low HRQoL scores.

**Conclusion:**

Among people with HIV, those with hypertension had lower HRQoL compared to those without. This calls for inclusion of quality-of-life assessment in the management of PWH who have been diagnosed with hypertension to identify those at risk and plan early interventions.

## Introduction

The scaling up of lifesaving antiretroviral therapy (ART) has led to a significant reduction in AIDS related mortality from 1.4 million in 2010 to 630,000 in 2022 [1] with sub-Saharan Africa (SSA) recording the highest reduction at 39.7% in 2019 [2]. As a result, the life expectancy of people with HIV (PWH) has substantially improved leading to an increase in the population of PWH over the age of 50 years [3]. This has coincided with an increase in the prevalence of age-related non-communicable diseases (NCDs) such as cardiovascular diseases among PWH [4]. Moreover, it is expected that over time, the prevalence of cardiovascular diseases among PWH in SSA is likely to increase due to the high predisposition [5, 6]. Hypertension is a known and important risk factor of cardiovascular diseases which is highly prevalent among PWH at 23.6–25.2% globally [7, 8] and at 8%—27.2% in Uganda [9–12].

The increasing incidence and prevalence of hypertension among PWH is likely to negatively affect the gains made in improving the health-related quality of life (HRQoL) of PWH through the provision of ART. In fact, the HRQoL of PWH greatly improves after initiation of ART [13] while individuals diagnosed with hypertension have been shown to have worse HRQoL when compared to their normotensive counterparts [14, 15]. Therefore, the possible HIV-hypertension comorbidity could significantly affect HRQoL. This could partly be attributed greater pill burden leading to sub-optimal adherence resulting into virological failure [16, 17]. The synergistic presence of risk factors for cardiovascular diseases such as alcohol use, smoking, and advanced age [18–20] as well as HIV associated stigma and adverse effects of ART [18, 21, 22] could be other probable causes of low HRQoL in this group.

The absence of evidence highlighting the effect of the HIV and hypertension on HRQoL makes the design of better target locally adapted HRQoL improving interventions difficult. This study compared HRQoL in PWH with and without hypertension in Uganda. The Medical Outcome Study (MOS-HIV) score, a disease specific HRQoL tool [23] and the EuroQol-5D- 5L (EQ-5D-5L), a generic HRQoL tool [24] which are likely to complement each other to generate HIV specific HRQoL and health utility scores [25] were used.

## Methods

### Study design

We conducted a comparative cross-sectional study between September 2020 and April 2021.

### Study setting

This study was conducted at the Infectious Diseases Institute (IDI) in Kampala, Uganda. The IDI is a center of excellence for HIV care and treatment with over 322,000 PWH under its care and over 70,000 patients recruited into its longitudinal cohorts [26]. IDI maintains an Antiretroviral therapy Long-Term (ALT) cohort which was established in 2014 with participants from urban communities in the central region of Uganda. The ALT is a prospective cohort of 1,000 PWH at the IDI clinic who have been on ART for over 10 years, enrolled at 1 year, and followed up yearly for 10 years. The detail methodology has been published elsewhere [27]. The cohort aims to determine the incidence of long-term drug side effects and toxicity, ART durability, and development of co-morbidities, with an emphasis on NCDs [27].

### Study population

We recruited PWH with or without hypertension who had been on ART for at least 10 years and were in the ALT cohort. The prolonged exposure to certain classes of ART (≥ 10 years) and HIV could increase their risk of developing hypertension hence their selection. Participants with hypertension were entered into this study based on the existing ALT Cohort standard operating procedure. The ALT Cohort standard operating procedures defined hypertension as three systolic measurements above 140 mm Hg or diastolic measurement above 90 mm Hg or history of treatment with antihypertensive drugs (as per ICEA) or patient-reported hypertension [27]. In addition, we included participants who were 18 years and above, were willing to give written informed consent, and willing to complete all study procedures. Pregnant women at the time of the study were excluded.

### Sampling technique

**Fig 1:**
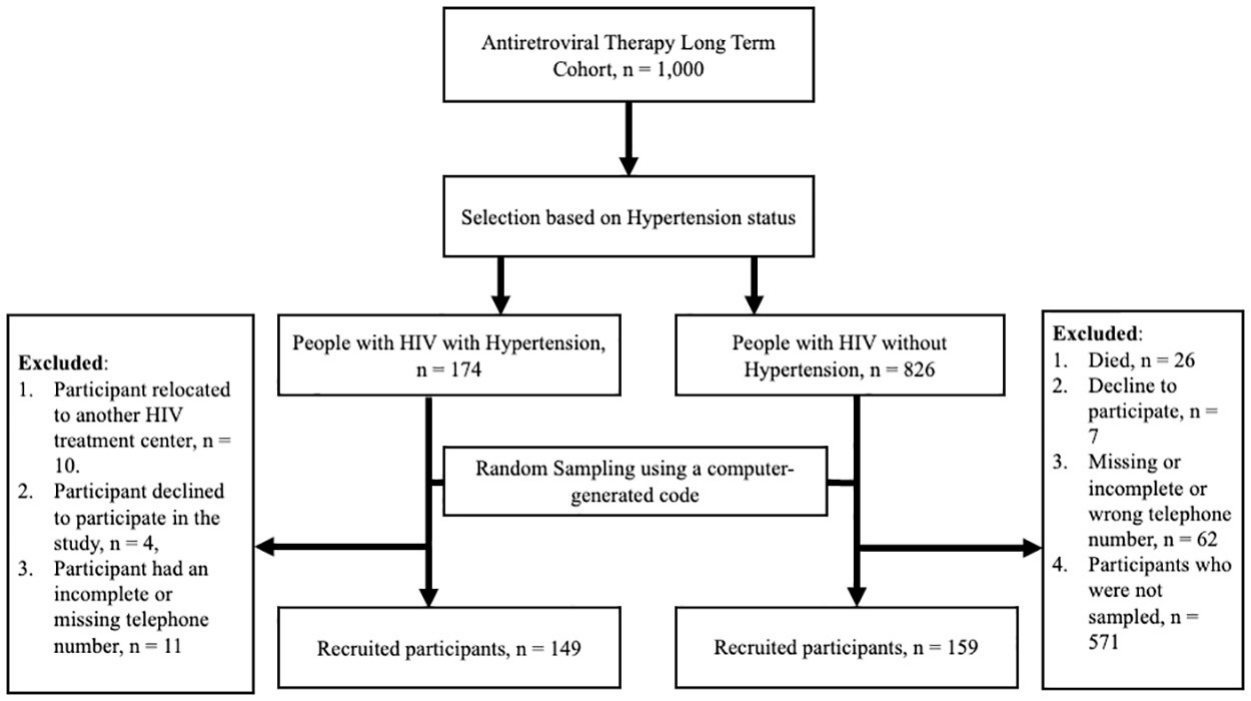
The flow chart representing how participants were selected in this study.

We obtained an electronic register of participants in the ALT cohort at IDI and performed stratified random sampling using computer generated random numbers based on the strata of presence or absence of confirmed hypertension at most recent follow up date for the participants (at most 6 months prior to our study). Hypertension was defined as either taking antihypertensives at the time of the last clinic visit and or elevated blood pressures of >140/90 mmHg as per the Uganda National guidelines at the time. Of these, 174 had a diagnosis of hypertension as determined by the ALT cohort’s clinical criteria and 826 did not have a diagnosis of hypertension (Fig 1). Due to smaller numbers of the hypertensive patients, all of them were subsequently considered while due to the large numbers only a subset was considered. Participants’ phone contact information were retrieved from the clinic database and research assistants made phone calls with the potential participants to provide study related information and schedule study visits. Stratified random sampling was chosen because it would provide an unbiased representative sample of the different subgroups under consideration for our study.

### Sample size estimation

We calculated the sample size using the formula of comparing means in two groups and a prior study conducted in Rwanda among HIV positive patients where the overall quality of life (irrespective of mental or physical health scores) was estimated at 50.4 ± 31.74 [19]. Due to absence of data comparing HRQoL among patients with HIV and HTN and those with HIV alone using the EQ-5D-5Lquestionnaire at the time of the study, we assumed a 10-point difference mean quality of life score points on the MOS-HIV questionnaire between the two groups at a power of 80% and type 1 error of 0.05. The more conservative 10-point difference in mean quality of life score points instead of the 16-point (derived from 0.5*SD, SD = 31.74) [28] clinically meaningful difference was chosen to further minimize the type 1 error rate. We needed 318 patients (159 participants in each group), assuming an equal distribution of participants in each group. We recruited 308/318 participants at IDI study site with all the 159 participants without hypertension recruited but 149/159 participants with hypertension were recruited giving us a power of 77.5%. Reasons for failure to reach the desired sample size in the latter category included death, failure to consent, being unreachable as shown in Fig 1.

### Data collection

At enrollment, we conducted face to face interviews with participants who consented to participate in the study using validated English or Luganda versions of the EQ-5D-5L and MOS-HIV questionnaires at a single sitting in a quiet room depending on the participants’ preference. Research assistants underwent training using both versions to ensure proper translation and interpretation of the questions. An electronic data collection tool, Kobo Toolbox [29], was used to collect data. Data such as ART duration, WHO clinical stage, viral load, diagnosis of opportunistic infections at the last follow up visit was extracted from the electronic database of the participants. HRQoL was measured using EQ-5D-5L, and disease-specific MOS-HIV tools.

### HRQoL measures

**EQ-5D-5L** [30, 31] is a standardized instrument that measures health status in 5 dimensions–Mobility, Self-Care, Usual Activities, Pain/Discomfort and Anxiety/Depression (EQ-5D). These are combined into a single utility score. The EQ-5D-5L also measures a respondent’s self-rated health on a linear visual analogue scale (EQ-VAS 20cm). At the time of the study (mid-year 2020) and analysis [32], there were no value sets available for the Ugandan population and the EQ-5D-5L had not been completely validated in Uganda. Additionally, no specific studies had been conducted in Uganda to evaluate the use of the EQ-5D-5L in chronic diseases like Hypertension and HIV patients. A single EQ-5D-5L utility score for our participants was computed based on a scoring algorithm from a general population survey in Zimbabwe [33]. The utility scores for the Zimbabwean population were appropriate for use in this valuation of health study because of absence of any other African population utility scores and the closeness of the population characteristics to the Ugandan population. In the foregoing Zimbabwe study, negative values for utility scores worse than death were generated by asking study respondents about how they valued a different set of eight randomly selected health states (either one or two of very mild, mild, moderate, and severe states) and the 33333 as the eighth state. Additionally, in this study, the research evaluated 38 different health states from the combinations of the five EQ-5D domains. The participants ranked states spent in perfect health for ten years as 11111 (example target state) [33] while states worse than death (10- target state) followed by the additional years in full health. Analysis of the self-administered EQ-5D-5L questionnaire and EQ5D visual analogue scale data revealed that nearly one third of participants had some or sever pain/discomfort and few people had problems in self-care, mobility, or usual activities. Higher VAS scores were reported among the Zimbabwean participants. The EQ-5D-5L’s performance based on studies done in the *Shona* and English languages in Zimbabwe had been documented with reliability kappa statistics ranging between 0.65 and 1.00 across all domains [33, 34]. In our study, a time trade-off (TTO) approach was used to value health states [30]. Utility scores were got by subtracting one minus disutility based on methods in a study conducted in Ethiopia [35]. The **MOS-HIV** is a 35-item tool that assesses the ten dimensions of heath in individuals living with HIV [36]. It provides overall physical and mental health summary scores that are transformed or standardized to make comparisons among various dimensions that may have different response categories. The MOS-HIV questionnaire has been validated and used in African settings including Rakai in Uganda [19, 37]. The MOS- HIV health survey has 11 dimensions: Health perceptions (5 items), Physical function (6 items); Social function (1 item); Role function (2 items); Cognitive function (4 items); Pain (2 items); Vitality/energy (4 items); Mental health (5 items); Health distress (4 items); Quality of life (1 item); and Health transition (1 item) [23, 38]. The dimensions were represented as health perceptions (Question 1 and 11), bodily pain (question 2 and 3), physical function (question 4), role function (question 5 and 6), social function (question 7), mental health (question 8), vitality/energy (first 4 of question 9), health distress (last 4 of question 9), cognitive function (question 10), quality of life (question 12), and health transition (question 13). The team sought permission to use this tool in the study. Responses were aggregated and converted to a 0–100 point scale, with 100 representing the best health status and 0 the worst state of health as published elsewhere [39].

### Data analysis

Data were cleaned, coded, and analyzed using Stata 15.0. Descriptive statistics such as mean, standard deviations, proportions and interquartile ranges were used to describe the study population. The summary of the participants’ responses on a five-point Likert scale of severity for each of the domains of the EQ-5D-5L questionnaire were presented as proportions. Chi square and Fisher’s exact tests used to compare values between the non-hypertensive and HIV/hypertension group. The primary outcome was the mean EQ-5D-5L utility score of the study participants. Participant data were manually entered into the EQ-5D-5L five-level crosswalk utility index value calculator as described by the user manual [40, 41]. An EQ-5DL-5L profile was then built for each participant using the calculator algorithm for value sets from the Zimbabwean population [33] and utility scores were calculated based on the Ethiopian population [35]. The HRQoL scores based on the EQ-5D-5L questionnaire were compared by the different viral load copies of ≤1000 or > 1000 using the Fisher’s exact and Mann-Whitney U tests. Questionnaire domains, physical health and mental health summaries were calculated from the MOS-HIV dimensions [42]. The component summary scores (physical and mental health summary scores) were then standardized so that each had a mean of 50 and a standard deviation of 10 [43]. Unadjusted differences in demographic variables and EQ-5D-5L/ MOS-HIV scores between the patients with HIV and hypertension and those without hypertension were assessed using Wilcoxon rank sum tests (for skewed distributions) or student t test (normally distributed) for continuous variables. The Chi square test was used to compare the categorical variables. A linear regression model was fitted by considering the EQ-5D-5L utility scores as the dependent variable. Covariates such as age, sex, hypertension and other social demographic characteristics were then added to the multiple linear regression model as done in a previous study [44]. The models were tested for normality, heteroscedasticity, linearity, and independence of observations. We included factors with a p-value less than 0.20 or clinical significance and known associations from prior literature in the multivariable model [45]. The unadjusted and adjusted coefficients with their 95% confidence intervals were presented.

### Ethics statement

The study was approved by the Makerere University School of Biomedical Sciences Research and Ethics Committee (SBS 750), University of Washington Institutional Review Board (STUDY0000974) and Uganda National Council for Science and Technology approval number HS581ES. The study was done in accordance with the ethical guidelines of the 1975 Declaration of Helsinki and principles of good clinical practice. Participants were informed about the purpose and procedure of the study. Written and verbal informed consent was obtained before enrollment into the study. No participant identifier information was obtained.

## Results

We recruited 308 participants of whom 149 individuals had hypertension and HIV while 159 participants were without hypertension (only had HIV). Fig 1 shows how participants were selected to take part in the study.

### Participant characteristics

Of the 308 participants recruited, 190 (61.7%) were females. Participants with hypertension were older than those without hypertension (Mean ± SD: 53.7± 8.3 years vs 49.9 ± 8.6 years). Table 1 highlights more details on the socio-demographic characteristics of the participants.

**Table 1:** The socio-demographic and clinical characteristics of participants recruited into this study.

Characteristic	Hypertension, n (%)	No Hypertension, n (%)
**Age in completed years**		
Mean ± Standard Deviation (SD)	53.7 ± 8.3 years	49.9 ± 8.6 years
Range	38–78	31–73
**Sex**		
Female	86 (57.7)	104 (65.4)
Male	63 (42.3)	55 (34.6)
**Marital status**		
Divorced	5 (3.4)	11 (6.9)
Married	51 (34.2)	46 (28.9)
Single	33 (22.2)	49 (30.8)
Cohabiting	21 (14.1)	25 (15.7)
Widow/widowed	39 (26.2)	28 (17.6)
**Occupation**		
None	18 (12.1)	18 (11.3)
Peasant farmer	23 (15.4)	25 (15.7)
Housewife	5 (3.4)	4 (2.5)
Salaried employee	23 (15.4)	23 (14.5)
Self employed	74 (49.7)	80 (50.3)
Other (pastor, pensioner)	6 (4.0)	9 (5.7)
**Monthly income in Uganda shillings in 10^3**		
Median (Interquartile Range (IQR))	100 (0–450)	40 (0–250)
**Level of education**		
No formal education	6 (4.0)	14 (8.8)
Primary	52 (34.9)	67 (42.2)
Secondary	69 (46.3)	58 (36.5)
University	4 (2.7)	3 (1.9)
Other tertiary institution	18 (12.1)	17 (10.7)
**Duration of ART in years**		
Median (IQR)	15.35 (14.89–15.79) years	15.32 (14.94–15.87) years
**Latest World Health Organization (WHO) stage**		
I		
II	19 (12.8)	17 (10.8)
III	77 (52.0)	75 (47.5)
IV	52 (35.1)	66 (41.8)
**Presence of treatment supporter**		
No	50 (33.6)	54 (34.0)
Yes	99 (66.4)	99 (66.0)
**Viral load at last follow-up visit (copies per ml)** ^a^		
≤1000	153 (96.3)	145 (97.3)
> 1000	6 (3.8)	4 (2.7)
**Cardiovascular event at last follow-up**		
No	146 (98.7)	158 (100.0)
Yes	2 (1.3)	0 (0.0)
**Presence of opportunistic infection at last follow up visit** ^a^		
Yes ^b^	0 (0.0)	2 (1.3)
No	148 (100.0)	156 (98.7)
**Knowledge of the partner’s HIV status**		
No	58 (38.9)	61 (38.4)
Yes	91 (61.1)	98 (61.6)
**Current use of contraceptives**		
No	86 (57.7)	92 (57.9)
Yes	63 (42.3)	67 (42.1)
**History of smoking**		
No	114 (76.5)	138 (86.8)
Yes	35 (23.5)	21 (13.2)
**History of alcohol intake**		
No	125 (83.9)	135 (84.9)
Yes	24 (16.1)	24 (15.1)

^a^ Virological failure classification based on the *Consolidated guidelines on the use of antiretroviral drugs for treating and preventing HIV infection*: *recommendations for a public health approach*. World Health Organization, 2016.

^b^ Only opportunistic infection present was tuberculosis.

WHO- World Health Organization, SDA- Seventh Day Adventist.

Concerning the clinical history of the participants, the median duration of ART was similar for both participants with or without hypertension at 15.35 (14.89–15.79) years and 15.32 (14.94–15.87) years respectively. More participants without hypertension were in stage IV of the WHO HIV staging compared to those with hypertension (41.8% vs 35.1%, respectively). Ninety-nine participants with or without hypertension had a treatment supporter.

Regarding risk factors of cardiovascular disease, only 2 (1.3%) of participants with hypertension had a history of cardiovascular event at their last follow-up. Thirty-five (23.5%) PWH with hypertension had a history of smoking while only 21 (13%) PWH without hypertension had such history. The same number of participants with hypertension or without hypertension had a history of alcohol intake, 24.

### HRQoL scores

Based on the EQ-5D-5L utility scores (Table 2), the median utility score from the participants was 0.73 (0.38–0.80). Participants with hypertension had lower median utility scores compared to participants without hypertension (0.71 (0.33–0.80) vs 0.80 (0.44–0.80), p value = 0.029). Close to three quarters of the participants, 223 (72.4%), had moderate problems with mobility with more participants without hypertension reporting this issue than those without hypertension (131 (82.4%) VS 92 (61.7%), p value < 0.001). In addition, a higher proportion of participants with hypertension had extreme problems, 23 (15.4%) as compared to those without hypertension, 8 (5%), p value < 0.001. Fifty-four (17.5%) participants had extreme anxiety/ depression. Having hypertension was associated with higher proportions of moderate or severe or extreme problems as compared to those without hypertension, p value < 0.05.

**Table 2:** The proportion of participants who had different HRQoL scores based on the EQ-5D-5L questionnaire.

Characteristic	Overall*, n (%)	Hypertension, n (%)	No Hypertension, n (%)	P value
**Mobility**				
No problem	2 (0.6)	0 (0.0)	2(1.3)	<0.001**
Slight problem	41 (13.3)	26 (17.4)	15 (9.4)	
Moderate problem	223 (72.4)	92 (61.7)	131(82.4)	
Severe problem	11 (3.6)	3 (1.9)	8 (5.4)	
Extreme problem	31 (10.1)	23 (15.4)	8 (5.0)	
**Self care**				
No problem	1 (0.3)	0 (0.0)	1 (0.6)	0.153**
Slight problem	8 (2.6)	2 (1.3)	6 (3.8)	
Moderate problem	290 (94.2)	140 (94.0)	150 (94.3)	
Severe problem	2 (0.7)	2 (1.3)	0 (0.0)	
Extreme problem	7 (2.3)	5 (3.4)	2 (1.3)	
**Usual activities**				
No problem	5 (1.6)	2 (1.3)	3 (1.9)	0.574**
Slight problem	20 (6.5)	11(7.4)	9 (5.7)	
Moderate problem	270 (87.7)	127 (85.2)	143 (89.9)	
Severe problem	3 (1.0)	2 (1.3)	1 (0.6)	
Extreme problem	10 (3.2)	7 (4.7)	3 (1.9)	
**Pain/discomfort**				
No problem	6 (1.9)	2 (1.3)	4 (2.5)	0.156**
Slight problem	63 (20.4)	38 (25.5)	25 (15.7)	
Moderate problem	157 (51.0)	67 (45.0)	90 (56.6)	
Severe problem	23 (7.5)	11(7.4)	12 (7.6)	
Extreme problem	59 (19.2)	31 (20.8)	28 (17.6)	
**Anxiety/Depression**				
No problem	186 (60.4)	76 (51.0)	110 (69.2)	0.007**
Slight problem	6 (1.9)	4 (2.7)	2 (1.3)	
Moderate problem	50 (16.2)	34 (22.8)	16 (10.1)	
Severe problem	12 (3.9)	7 (4.7)	5 (3.1)	
Extreme problem	54 (17.5)	28 (18.8)	26 (16.4)	
EQ5D5L utility index scores***, **median and Interquartile range**	0.73 (0.38–0.80)	0.71 (0.33–0.80)	0.80 (0.44–0.80)	0.029*****
Visual analog scale (VAS) score****, **median and Interquartile range**	80 (65–90)	75 (60–90)	80 (70–90)	0.241*****

*Overall (entire dataset of 308 is considered without categorizations)

**P value based on Fisher’s exact test

*** EQ-5D-5L utility index ranges from 0 (worst HRQOL) to 1 (best HRQOL)

****VAS score ranges from 0 = worst health to 100 = best Health

*****P value based on the Mann Whitney U test because of unequal variances.

Regarding MOS-HIV scores (Table 3), participants with hypertension had lower mean overall physical health scores compared to participants without hypertension (48.44 ± 10.17 VS 51.44 ± 9.65, p value = 0.008). The overall mean score was lowest in the physical function domain, 18.67 ± 21.39. In general health perception domain, participants without hypertension had a lower mean score than those with hypertension (26.54 ±17.82 Vs 30.76 ± 22.95) but there was no statistical significance (p = 0.072). Role functioning dimension had the biggest point difference in the physical health domain between the two sets of participants (9.66, 95% Confidence Interval (CI) (2.62–16.71)), with participants with hypertension having a lower score in this domain than those without hypertension (75.36 ± 33.75 VS 85 ± 29.03, p value = 0.007). In the mental health domain, the overall mean mental health summary score was 50 ± 10 with participants with hypertension having a higher overall mean mental health summary score (50.62 ± 9.75) than those without hypertension (49.42 ± 10.21). Generally, participants with hypertension had better scores than those without hypertension in majority of the components, however, none of the components were statistically significant.

**Table 3:** The mean scores of HRQoL scores based on MOS-HIV.

MOSHIV dimensions	Overall, mean ± SD	Minimum	Maximum	Hypertension, mean ± SD	No hypertension, mean ± SD	Mean difference between Hypertension and non-Hypertension (95% CI)	Value***
**Physical health summary**	**50 ± 10**	**15.02**	**62.06**	**48.44 ± 10.17**	**51.44 ± 9.65**	**3.01 (0.78–5.23)**	**0.008**
General health perceptions	28.57 ± 20.52	0	84.21	30.76 ± 22.95	26.54 ± 17.82	-4.21 (-8.80–0.37)	0.072
Physical functioning	18.67 ± 21.39	0	91.67	20.66 ± 20.95	16.82 ± 21.71	-3.84 (-8.63–0.95)	0.116
Role functioning	80.36 ± 31.71	0	100	75.36 ± 33.75	85.00 ± 29.03	9.66 (2.62–16.71)	**0.007**
Social functioning	73.25 ± 22.39	0	100	73.48 ± 24.13	73.63 ± 20.73	0.79(-4.24–5.82)	0.756
Bodily pain	52.38± 18.80	0	100	50.52± 19.19	54.09 ± 18.32	3.57 (-0.63–7.78)	0.096
**Mental health summary**	**50 ± 10**	**13.61**	**70.64**	**50.62 ± 9.75**	**49.42 ± 10.21**	**-1.19 (-3.44–1.05)**	**0.295**
Cognitive functioning	69.62 ± 18.95	0	100	71.58 ± 16.75	67.81 ± 20.68	-3.78 (-8.01–0.46)	0.081
Mental health	63.35± 17.10	0	100	65.02 ± 18.17	61.80 ± 15.94	-3.22 (-7.05–0.59)	0.098
Vitality/energy/fatigue	59.14 ± 18.45	0	100	59.89 ± 18.58	58.43 ± 18.54	-1.46 (-5.63–2.70)	0.491
Health distress	71.93 ± 17.59	0	100	70.37 ± 20.00	73.38 ± 14.94	3.00 (-0.94–6.94)	0.135
Quality of life	36.61 ± 31.66	0	100	34.79 ± 30.39	38.28 ± 32.81	3.48 (-3.62–10.59)	0.335
Health transitions	28.41 ± 31.01	0	100	26.52 ± 31.03	30.16 ± 30.98	3.63 (-3.32–10.59)	0.305

***P values based on comparing the means between the two different groups.

#### Factors associated with low HRQoL scores based on EQ-5D-5L utility scores

Based on a multiple linear regression (Table 4), having an income > 70,000 Uganda shillings was associated with a 0.05 units reduction in HRQoL scores as compared to those earning less ≤ 70,000 Uganda shillings (Adjusted β = -0.049, 95% CI, -0.097—(-0.001), p value = 0.044). The presence of a history of hypertension led to 0.040 reduction in the HRQoL utility scores as compared to those without hypertension (Adjusted β = -0.040, 95% CI -0.074—(-0.005), p value = 0.023). Participants who had disclosed their HIV status to their partners had a 0.065 reduction in HRQoL scores as compared to those who had not (Adjusted β = -0.065, 95% CI, -0.122—(-0.008). Those participants who had a history of smoking had a 0.053 reduction in the HRQoL scores as compared to those who had no history of smoking (Adjusted β = -0.053, 95% CI, -0.100—(-0.005), p value = 0.029). The 0.05 reduction may have uncertain clinical significance to the participants.

**Table 4:** The factors associated with low HRQoL scores based on EQ-5-D5L utility scores.

Characteristic	Un adjusted coefficients, β (95% Confidence intervals)	P value	Adjusted coefficients, β (95% Confidence intervals)	P value
**Hypertension**				
No	Reference		Reference	
Yes	-0.042(-0.076—(-0.007))	0.017	-0.040 (-0.074—(-0.005))	0.023
**Marital**				
Married	Reference			
Single	0.036(-0.006–0.077)	0.095		
Widowed	0.023 (-0.021–0.068)	0.305		
Divorced	-0.034(-0.114–0.046)			
**Education level**				
Primary or no formal education	Reference			
Secondary	-0.019(-0.056–0.018)	0.321		
University or tertiary	-0.017 (-0.071–0.036)	0.514		
**Treatment supporter**				
No	Reference		Reference	
Yes	0.012(-0.024–0.049)	0.497	0.015(-0.021–0.053)	0.405
**Income status per month (in Uganda Shillings X10^3)**				
≤70	Reference		Reference	
71–210	-0.017(-0.001–0.076)	0.499	-0.049(-0.097—(-0.001))	0.044
>210	0.037(-0.069–0.034)	0.058	-0.043(-0.084—(-0.002))	0.038
**Sex**				
Male	Reference		Reference	
Female	-0.007(-0.042–0.029)	0.711	-0.043(-0.085–0.000)	0.050
**Duration of ART**				
>15 year	Reference			
≤15 year	-0.001(-0.039–0.037)	0.953		
**Do you know your partners HIV status**				
No	Reference		Reference	
Yes	0.004 (-0.032–0.039)	0.839	-0.51(-0.007–0.108)	0.086
**Has the patient disclosed their HIV status to partner**				
No	Reference		Reference	
Yes	-0.018(-0.053–0.017)	0.314	-0.065(-0.122—(-0.008))	0.026
**Latest WHO stage**				
II	Reference			
III	0.014(-0.042–0.071)	0.617		
IV	0.01(-0.05–0.06)	0.849		
**Age**	-0.001 (-0.005–0.003)	0.509		
**Currently smoke**				
No	Reference		Reference	
Yes	-0.051(-0.096—(-0.007))	0.024	-0.053(-0.100—(-0.005))	0.02
**Currently drink alcohol**				
No	Reference			
Yes	-0.029 (-0.076–0.018)	0.235		

## Discussion

In this cross-sectional study, we found that hypertensive participants had lower mean utility scores than those without hypertension based on EQ-5D-5L tool. Whereas participants with hypertension had better mental health scores than their counterparts, their physical health scores were worse than those without hypertension. After adjusting for confounders, hypertension, higher income status, disclosure of the HIV status of the participants to their participants and history of smoking were associated with low EQ-5D-5L utility scores.

There is limited literature comparing the health-related quality of life of HIV positive individuals with hypertension and those without. However, studies assessing the quality of life of PWH have shown that PWH on ART have improved quality of life [19, 46] which is consistent with our findings. On the other hand, participants with hypertension had lower scores in most of the components especially in the physical health summary components which is similar to what was reported in other settings [19, 47]. Although there was no significant difference in the mental health scores in both groups, participants had lower mental health scores overall. This has also been reported in Rwanda [19], Uganda [37] and Ethiopia [47].

Our findings suggest that hypertension reduces the quality of life among PWH. Schenker and colleagues showed that the presence of chronic co-morbidities as well as polypharmacy in individuals with advanced age led to poor quality of life and increased symptom burden [48]. In our study, individuals with hypertension were slightly older than those without and had poorer physical health scores which could have had a negative effect on their ability to carry out their day-to-day activities leading to the differences in the physical health summary scores, mobility, and anxiety/depression scores. Another study done in Malawi found that hypertensive PWH had poor adherence to their anti-hypertensive medication, difficulty in adjusting their lifestyles, and the costs involved in purchasing the medicine, which may have further contributed to poor HRQoL scores. Whereas mental health scores were not statistically significant, they were generally low–possibly due to the high prevalence of psychological disorders in HIV positive individuals and the social stigma they face [49, 50]. In addition, studies have shown that having a treatment supporter is associated with improved quality of life especially mental health through the provision of social support [51]. Much as the study was done among participants who have been on long term ART (> 10 years) and almost all participants had treatment supporters, our study found that the disclosure of HIV/AIDS status to partners was associated with low HRQoL scores. This could be attributed to the rejection they faced or are still facing from their partners [52] causing further distress. Therefore, subsequent studies should explore how stigma and disclosure of HIV status could affect HRQoL of HIV positive individuals who have been on long term ART.

Co-morbidity with hypertension, history of smoking, and higher income status was associated with low HRQoL. Participants with hypertension have a high risk of developing cardiovascular diseases which is likely to cause significant morbidity including limitations in performing their daily physical intensive activities hence the poor score in the role functioning domain. Unlike a study done in China [53], a higher income status was associated with low HRQoL scores in this study. This could be due to stress from the closure of businesses and distortion in economic activities because of the coronavirus disease lockdown. The lockdown restriction of movements prevented people with chronic diseases such as HIV and hypertension from accessing their clinics, leading to disruptions in the supply of critical medications such as ART and antihypertensives [54]. This situation, coupled with financial disruptions, adversely affected mental health, increasing the risk of anxiety and depression in PWH with or without hypertension. History of smoking was associated with a reduction in HRQoL which may be explained by the increase in evidence suggesting that smoking has a negative impact on the psychological wellbeing of individuals and less likely to seek social support and have poor adherence to ART as well as more likely to experience more social stigma and develop other co-morbidities such as liver cirrhosis and cancers hence affecting their HRQoL [55].

Our study has limitations. First, the EQ-5D-5L has not been validated in a Ugandan population. Secondly, we were unable to collect data on co-morbidities such as obesity, diabetes, hypertension treatment and pocket costs that could have impacted results. Therefore, future studies should explore how these factors affect the HRQoL of PWH with or with hypertension. Thirdly, there was a significant age difference between PWH with hypertension and those without. Lastly, the study was conducted among patients who have been on ART ≥10 years receiving care from a center of excellence in HIV care where they receive high quality care. This population may not be representative of all individuals living with HIV. Despite these limitations, the proportion of ageing PWH in Uganda is progressively increasing which highlights the need to evaluate the changes in functionality, monitor treatment outcomes and effects on patients, and changes in care because of multimorbidity. Future longitudinal studies should explore the differences and changes in HRQoL scores of PWH with co-morbidities in rural and urban settings due to the differences in lifestyles.

## Conclusion

Our findings provide evidence on the potential effect of hypertension on the HRQoL of PWH and illustrate the need for strategies targeting the improvement of the HRQoL of PWH on long term ART with or without hypertension. Further research on how HRQoL affects treatment outcomes of PWH diagnosed with hypertension compared to those without hypertension is warranted.

HIV/AIDS programs should incorporate hypertension-related screening services into routine clinic as well as strengthen patient education on hypertension and its risk factors to foster early management of hypertension.

## Abbreviations

ALT: ART Long Term
ART: Antiretroviral Therapy
EQ- 5D- 5L: European Quality of Life 5 Dimensions 5 levels
HRQoL: Health Related Quality of Life
MOS-HIV: Medical Outcomes Study HIV Health Survey
NCDs: Non-Communicable Diseases
WHO: World Health Organization

## References

[1] UNAIDS. Global HIV & AIDS statistics—Fact sheet Gevena: UNAIDS; 2023 [cited 2023 20th February]. https://www.unaids.org/en/resources/fact-sheet.

[2] Jahagirdar D, Walters MK, Novotney A, Brewer ED, Frank TD, Carter A, et al. Global, regional, and national sex-specific burden and control of the HIV epidemic, 1990–2019, for 204 countries and territories: the Global Burden of Diseases Study 2019. The Lancet HIV. 2021;8(10):e633–e51. doi: 10.1016/S2352-3018(21)00152-1 34592142 PMC8491452

[3] Haeuser E, Serfes AL, Cork MA, Yang M, Abbastabar H, Abhilash E, et al. Mapping age-and sex-specific HIV prevalence in adults in sub-Saharan Africa, 2000–2018. BMC medicine. 2022;20(1):1–24. 36529768 10.1186/s12916-022-02639-z PMC9760541

[4] Chang D, Esber AL, Dear NF, Iroezindu M, Bahemana E, Kibuuka H, et al. Non‐communicable diseases by age strata in people living with and without HIV in four African countries. Journal of the International AIDS Society. 2022;25:e25985. doi: 10.1002/jia2.25985 36176018 PMC9523000

[5] Shah ASV, Stelzle D, Lee KK, Beck EJ, Alam S, Clifford S, et al. Global Burden of Atherosclerotic Cardiovascular Disease in People Living With HIV. 2018;138(11):1100–12. doi: 10.1161/CIRCULATIONAHA.117.033369 29967196 PMC6221183

[6] Ake JA, Polyak CS, Crowell TA, Kiweewa F, Semwogerere M, Maganga L, et al. Noninfectious comorbidity in the African cohort study. Clinical Infectious Diseases. 2019;69(4):639–47. doi: 10.1093/cid/ciy981 30476001 PMC6669288

[7] Xu Y, Chen X, Wang K. Global prevalence of hypertension among people living with HIV: a systematic review and meta-analysis. Journal of the American Society of Hypertension. 2017;11(8):530–40. doi: 10.1016/j.jash.2017.06.004 28689734

[8] Bigna JJ, Ndoadoumgue AL, Nansseu JR, Tochie JN, Nyaga UF, Nkeck JR, et al. Global burden of hypertension among people living with HIV in the era of increased life expectancy: a systematic review and meta-analysis. J Hypertens. 2020;38(9):1659–68. doi: 10.1097/HJH.0000000000002446 32371769

[9] Sander LD, Newell K, Ssebbowa P, Serwadda D, Quinn TC, Gray RH, et al. Hypertension, cardiovascular risk factors and antihypertensive medication utilisation among HIV‐infected individuals in R akai, U ganda. Tropical Medicine & International Health. 2015;20(3):391–6. 25430847 10.1111/tmi.12443 PMC4308448

[10] Niwaha AJ, Wosu AC, Kayongo A, Batte C, Siddharthan T, Kalyesubula R, et al. Association between blood pressure and HIV status in rural Uganda: Results of cross-sectional analysis. 2021;16(1). 10.5334/gh.858 PMC7880004 33598392

[11] Kansiime S, Mwesigire D, Mugerwa H JPo. Prevalence of non-communicable diseases among HIV positive patients on antiretroviral therapy at joint clinical research centre, Lubowa, Uganda. 2019;14(8):e0221022. doi: 10.1371/journal.pone.0221022 31398244 PMC6688817

[12] Kasoma Mutebi R, Weil Semulimi A, Mukisa J, Namusobya M, Namirembe JC, Nalugga EA, et al. Prevalence of and factors Associated with Hypertension among adults on Dolutegravir-Based antiretroviral therapy in Uganda: A Cross Sectional Study. Integrated Blood Pressure Control. 2023:11–21. doi: 10.2147/IBPC.S403023 37102123 PMC10123006

[13] Dutra BS, Lédo AP, Lins-Kusterer L, Luz E, Prieto IR, Brites C. Changes health-related quality of life in HIV-infected patients following initiation of antiretroviral therapy: a longitudinal study. Brazilian Journal of Infectious Diseases. 2019;23:211–7. doi: 10.1016/j.bjid.2019.06.005 31344351 PMC9428026

[14] Adedapo ADA, Akunne OO, Adedokun BO. Comparative assessment of determinants of health-related quality of life in hypertensive patients and normal population in south-west Nigeria. Int J Clin Pharmacol Ther. 2015;53(3):265–71. doi: 10.5414/CP202257 . 25613540 PMC6102563

[15] Trevisol DJ, Moreira LB, Kerkhoff A, Fuchs SC, Fuchs FD JJoh. Health-related quality of life and hypertension: a systematic review and meta-analysis of observational studies. 2011;29(2):179–88. doi: 10.1097/HJH.0b013e328340d76f 21045726

[16] Nachega JB, Parienti J-J, Uthman OA, Gross R, Dowdy DW, Sax PE, et al. Lower pill burden and once-daily antiretroviral treatment regimens for HIV infection: A meta-analysis of randomized controlled trials. Clin Infect Dis. 2014;58(9):1297–307. Epub 2014/01/22. doi: 10.1093/cid/ciu046 . 24457345 PMC3982838

[17] Torres TS, Harrison LJ, La Rosa AM, Zheng L, Cardoso SW, Ulaya G, et al. Poor quality of life and incomplete self-reported adherence predict second-line ART virological failure in resource-limited settings. AIDS Care. 2021;33(10):1340–9. Epub 2021/01/26. doi: 10.1080/09540121.2021.1874275 . 33487029 PMC8298588

[18] Mohammed SA, Yitafr MG, Workneh BD, Hailu AD. Health-related quality of life and associated factors among people living with human immunodeficiency virus on highly active antiretroviral therapy in North East Ethiopia: Cross-sectional study. PLOS ONE. 2021;16(3):e0247777. doi: 10.1371/journal.pone.0247777 33667245 PMC7935299

[19] Biraguma J, Mutimura E, Frantz JM. Health-related quality of life and associated factors in adults living with HIV in Rwanda. SAHARA: Journal of Social Aspects of HIV / AIDS Research Alliance. 2018;15(1):110–20. doi: 10.1080/17290376.2018.1520144 30200815 PMC6136357

[20] Tüzün H, Aycan S, İlhan MN JCEjoph. Impact of comorbidity and socioeconomic status on quality of life in patients with chronic diseases who attend primary health care centres. 2015;23(3):188–94. doi: 10.21101/cejph.a3990 26615648

[21] Parcesepe AM, Nash D, Tymejczyk O, Reidy W, Kulkarni SG, Elul B. Gender, HIV-Related Stigma, and Health-Related Quality of Life Among Adults Enrolling in HIV Care in Tanzania. AIDS and behavior. 2020;24(1):142–50. doi: 10.1007/s10461-019-02480-1 . 30927114 PMC6768763

[22] Mutabazi-Mwesigire D, Katamba A, Martin F, Seeley J, Wu AW. Factors That Affect Quality of Life among People Living with HIV Attending an Urban Clinic in Uganda: A Cohort Study. PloS one. 2015;10(6):e0126810–e. doi: 10.1371/journal.pone.0126810 . 26039733 PMC4454695

[23] Wu AW, Revicki D, Jacobson D, Malitz F JQolr. Evidence for reliability, validity and usefulness of the Medical Outcomes Study HIV Health Survey (MOS-HIV). 1997;6(6):481–93. doi: 10.1023/a:1018451930750 9330549

[24] Euroqol. EQ-5D-5L. http://wwweuroqolorg/eq-5d-products/eq-5d-5lhtml

[25] Wu AW, Hanson KA, Harding G, Haider S, Tawadrous M, Khachatryan A, et al. Responsiveness of the MOS-HIV and EQ-5D in HIV-infected adults receiving antiretroviral therapies. Health and quality of life outcomes. 2013;11:42-. doi: 10.1186/1477-7525-11-42 . 23497257 PMC3602001

[26] IDI. Programs Kampala Infectious Diseases Institute; 2020 [cited 2021 24 July]. https://idi.mak.ac.ug/programs/research/.

[27] Castelnuovo B, Mubiru F, Kiragga AN, Musomba R, Mbabazi O, Gonza P, et al. Antiretroviral treatment Long-Term (ALT) cohort: a prospective cohort of 10 years of ART-experienced patients in Uganda. BMJ Open. 2018;8(2):e015490. doi: 10.1136/bmjopen-2016-015490 29467129 PMC5855467

[28] Faraone SV. Interpreting estimates of treatment effects: implications for managed care. Pharmacy and Therapeutics. 2008;33(12):700. 19750051 PMC2730804

[29] KoBoToolbox. Kobo Toolbox Cambridge: Havard Humanitarian Initiative; 2012 [cited 2021 24 July]. https://www.kobotoolbox.org/.

[30] Jelsma J, Ferguson G. The determinants of self-reported health-related quality of life in a culturally and socially diverse South African community. Bulletin of the World Health organization. 2004;82:206–12. 15112009 PMC2585936

[31] Group TE JHp. EuroQol-a new facility for the measurement of health-related quality of life. 1990;16(3):199–208. doi: 10.1016/0168-8510(90)90421-9 10109801

[32] Yang F, Katumba KR, Roudijk B, Yang Z, Revill P, Griffin S, et al. Developing the EQ-5D-5L value set for Uganda using the ‘lite’protocol. Pharmacoeconomics. 2022:1–13. doi: 10.1007/s40273-021-01101-x 34841471 PMC8627844

[33] Jelsma J, Hansen K, de Weerdt W, de Cock P, Kind P. How do Zimbabweans value health states? Population Health Metrics. 2003;1(1):11. doi: 10.1186/1478-7954-1-11 14678566 PMC317383

[34] Jelsma J, Mhundwa K, De Weerdt W, De Cock P, Chimera J, Chivaura V. The reliability of the Shona version of the EQ-5D. The Central African journal of medicine. 2001;47(1):8–13. doi: 10.4314/cajm.v47i1.8584 11961858

[35] Welie AG, Gebretekle GB, Stolk E, Mukuria C, Krahn MD, Enquoselassie F, et al. Valuing health state: an EQ-5D-5L value set for Ethiopians. 2020;22:7–14. 10.1016/j.vhri.2019.08.475 31683254

[36] Wu AW, Revicki DA, Jacobson D, Malitz FE. Evidence for reliability, validity and usefulness of the Medical Outcomes Study HIV Health Survey (MOS-HIV). Quality of life research: an international journal of quality of life aspects of treatment, care and rehabilitation. 1997;6(6):481–93. Epub 1997/08/01. doi: 10.1023/a:1018451930750 . 9330549

[37] Mast TC, Kigozi G, Wabwire-Mangen F, Black R, Sewankambo N, Serwadda D, et al. Measuring quality of life among HIV-infected women using a culturally adapted questionnaire in Rakai district, Uganda 2004. 81–94 p. 10.1080/09540120310001633994 14660146

[38] Revicki DA, Sorensen S, Wu AW JMc. Reliability and validity of physical and mental health summary scores from the Medical Outcomes Study HIV Health Survey. 1998:126–37. doi: 10.1097/00005650-199802000-00003 9475468

[39] Murri R, Fantoni M, Del Borgo C, Visona R, Barracco A, Zambelli A, et al. Determinants of health-related quality of life in HIV-infected patients. 2003;15(4):581–90. 10.1080/0954012031000134818 14509872

[40] EuroQoL. EQ-5D-5L User Guide Basic information on how to use the EQ-5D-5L instrument. The Netherlands: EuroQol Research Foundation; 2015 [cited 2021 April 5th]. http://www.euroqol.org/fileadmin/user_upload/Documenten/PDF/Folders_Flyers/EQ-5D-5L_UserGuide_2015.pdf.

[41] Van Hout B, Janssen M, Feng Y-S, Kohlmann T, Busschbach J, Golicki D, et al. Interim scoring for the EQ-5D-5L: mapping the EQ-5D-5L to EQ-5D-3L value sets. Value in health. 2012;15(5):708–15. doi: 10.1016/j.jval.2012.02.008 22867780

[42] Sloan J, Novotny P, Loprinzi C, editors. Analyzing Quality of Life (QOL) Endpoints in Clinical Trials via the SAS System 1998.

[43] Ware J, Kosinski M, Keller S JAusm. SF-36 physical and mental health summary scales. 2001:1994.

[44] Liu K, He L, Tang X, Wang J, Li N, Wu Y, et al. Relationship between menopause and health-related quality of life in middle-aged Chinese women: a cross-sectional study. BMC Women’s Health. 2014;14(1):7. doi: 10.1186/1472-6874-14-7 24410885 PMC3893455

[45] Hosmer DW Jr. Model‐building strategies and methods for logistic regression. Applied logistic regression. 2013:89–151.

[46] Yaya I, Djalogue L, Patassi AA, Landoh DE, Assindo A, Nambiema A, et al. Health-related quality of life among people living with HIV/AIDS in Togo: individuals and contextual effects. BMC Research Notes. 2019;12(1):140. doi: 10.1186/s13104-019-4171-x 30876448 PMC6419817

[47] Melaku T, Mamo G, Chelkeba L, Chanie T JProm. Health-related quality of life among people living with human immunodeficiency virus on highly active antiretroviral therapy in Ethiopia: PROQOL-HIV based survey. 2020;11:73. doi: 10.2147/PROM.S239429 32184689 PMC7063799

[48] Schenker Y, Park SY, Jeong K, Pruskowski J, Kavalieratos D, Resick J, et al. Associations Between Polypharmacy, Symptom Burden, and Quality of Life in Patients with Advanced, Life-Limiting Illness. Journal of General Internal Medicine. 2019;34(4):559–66. doi: 10.1007/s11606-019-04837-7 30719645 PMC6445911

[49] Mohammed M, Mengistie B, Dessie Y, Godana WJ JACR. Prevalence of depression and associated factors among HIV patients seeking treatments in ART clinics at Harar Town, Eastern Ethiopia. 2015;6(474):2.

[50] Petrushkin H, Boardman J, Ovuga E JPB. Psychiatric disorders in HIV-positive individuals in urban Uganda. 2005;29(12):455–8.

[51] Bajunirwe F, Tisch DJ, King CH, Arts EJ, Debanne SM, Sethi AK JAc. Quality of life and social support among patients receiving antiretroviral therapy in Western Uganda. 2009;21(3):271–9. doi: 10.1080/09540120802241863 19280404

[52] Atuyambe LM, Ssegujja E, Ssali S, Tumwine C, Nekesa N, Nannungi A, et al. HIV/AIDS status disclosure increases support, behavioural change and, HIV prevention in the long term: a case for an Urban Clinic, Kampala, Uganda. BMC Health Services Research. 2014;14(1):276. doi: 10.1186/1472-6963-14-276 24950958 PMC4076501

[53] Zhang Y, Ou F, Gao S, Gao Q, Hu L, Liu Y. Effect of Low Income on Health-Related Quality of Life: A Cross-sectional Study in Northeast China. Asia Pacific Journal of Public Health. 2013;27(2):NP1013–NP25. doi: 10.1177/1010539513496839 24097919

[54] Komasawa M, Aung MN, Nsereko C, Ssekitoleko R, Isono M, Saito K, et al. Impact of Hospital Closure on Patients with Communicable and Non-Communicable Diseases During the COVID-19 Pandemic in Uganda: A Cross-Sectional and Mixed-Methods Study. Risk Management and Healthcare Policy. 2023:2593–607. doi: 10.2147/RMHP.S419969 38045563 PMC10691269

[55] Turner J, Page-Shafer K, Chin DP, Osmond D, Mossar M, Markstein L, et al. Adverse impact of cigarette smoking on dimensions of health-related quality of life in persons with HIV infection. 2001;15(12):615–24. 10.1089/108729101753354617 11788076

