# Supplementary material for: Health-related quality of life and hypertension in people with HIV on long-term antiretroviral therapy in Uganda
Source: PLoS One. 2024 Aug 8;19(8):e0306928. doi: 10.1371/journal.pone.0306928 (PMC11309503; doi:10.1371/journal.pone.0306928)
Supplement: Attachment — Submitted filename: Response to the reviewers comments.docx [file pone.0306928.s001.docx]

**Reviewer #1: This is a case control study, not a cross-sectional study. It must be described, analysed and interpreted as a case control study. The introduction misrepresents the literature and must be rewritten.**

Thank you for your comment. We would like to disagree with the reviewer regarding the design of the study. We believe that the study fits a cross-sectional design because the participants were selected based on the exposure (Hypertension) and not the outcome (HRQoL).

In addition, the introduction and literature has been revised to ensure coherence and re-alignment.

**Introduction**

**References 3 and 4 are a bit out of date. The text shows the dates for reference 3, but not for reference 4. When was that prediction made about PWH living for 26.7 more years after starting ART? I’m sure it’s longer now.**

Thank you for the comment. These statements have been revised, line 66- 69 and the references updated.

**The interpretation of reference 5 is wrong. Figure 3 in reference 5 shows about 60% of PWH in Zimbabwe will have one or more NCDs by 2035 (not 26%).**

Thank you for the comment. These statements have been revised, line 71- 72 and the references updated.

**Reference 6 is about the Netherlands. The setting is obviously crucial information, in terms of expected prevalence of NCDs. Both papers make the point that most of the risk of NCD isn’t attributable to HIV, it’s attributable to background prevalence, which is largely based on the age distribution of the population, which is completely different in Zimbabwe from the Netherlands. In other words, the prediction from reference 5 is much more relevant for your study than reference 6. Also, it would make more sense to report that in the Netherlands in 2030 84% of PWH are predicted to have at least one NCD, rather than focusing in on CVD.**

Thank you for the comment. The statement has been revised accordingly to reflect the African situation, line 72. The reference from the modelling study in Netherlands has been removed.

**Reference 7: where does the 11-23.9% prevalence of hypertension come from? The paper says 12.4%. Also note that this paper reported on diagnosed hypertension and pointed out the true prevalence is likely to be higher. The discussion cited 3 other papers, and their reported prevalences of hypertension in HIV were 10.2% (Magodoro), 28.5% (Dimala; half of sample was ART naive) and 11% (Kwarisiima).**

Thank you for the comment. The statement has been revised accordingly and references updated, line 73- 74.

I stopped checking references at this point. I’ve seen enough.

**You should probably cite Katherine Davis’ 2021 systematic review, “Association between HIV infection and hypertension: a global systematic review and meta-analysis of cross-sectional studies”, doi: 10.1186/s12916-021-01978-7. It shows how people with HIV have higher prevalence of hypertension than the population in North America and Europe, but lower prevalence in Africa.**

Thank you for your comment. It has been included.

**Methods**

**There was no reason to drop the 1 participant and reduce your sample size from 309 to 308. We have sample sizes so that we don’t waste patients’ time collecting unnecessary data. Once you have gone to the trouble of collecting that data, you should use it, not throw it out.**

Thank you for your comment. This additional participant was removed. Although exceeding the sample size had incomplete data for many key variables under study and therefore was dropped from the analysis. The flow diagram has been updated as a result.

**256 out of 826 people without hypertension were screened, correct? So that means this is a case control study. Cases were selected because they have hypertension, controls were selected because they don’t.**

Thank you for your comment. Whereas 256 out of 826 patients with hypertension were screened. The outcome of interest of this study was health related quality of life and not hypertension. Hypertension was considered as a major exposure variable and therefore, the assertion that this was a case control analysis does not hold based on Steve R Cummings Designing Clinical research page 109.

**Results**

**In Table 1, it isn’t appropriate to have a total column. The total is an artificial group of people, made up of 149 people with hypertension and 159 without. The characteristics of that group aren’t useful, because they are not a group, they’re two groups pushed together. Describe cases and controls separately in the text.**

Thank you for your comment. This has been addressed. However, the exposures are hypertension and no hypertension.

**“In the mobility domain, participants without hypertension had problems while in the anxiety and depression domain, having hypertension was associated with problems.” That isn’t true. 15% of participants with hypertension had extreme mobility problems compared to 5% of controls.**

Thank you for your comment. You are correct. The interpretation has been updated to reflect the meaning. It is now based upon the proportions of which the P-value is a chi square based.

**The cases are older and more likely to be male (of course – because they were selected for hypertension) and have higher income. Because they’re older they have worse mobility, worse selfcare, more pain. All this was inevitable from the study design.**

Thank you for your comment. This has been added as part of the discussion, line 277-279.

**The research question is whether controls have worse quality of life. They probably will because they’re older. If you want to know about the causal effect of hypertension on quality of life you’ll need to adjust for age, sex, income etc. On the other hand, if you just want to describe the needs and characteristics of people with HIV and hypertension, you shouldn’t adjust for those things because they are some of the reasons why people with hypertension might have worse quality of life.**

Thank you for your comment. Yes, we agree that if QOL is the outcome of interest, we should adjust for sex, age and income and use hypertension as the main exposure variable. This was the goal of this research study and we implemented this during the association analysis. Our goal is not to discuss the needs and characteristics of the people with HIV. However, this is described in the first results of the study population (Table 1). We adjust for them so that we can make proper predictions.

**Table 3: this analysis combines participants from the two groups, which isn’t appropriate. People with hypertension have higher viral load and worse mobility. Therefore, inevitably, viral load will be associated with mobility because of the choices in the study design. Look at cases and controls separately. Table 4 has the same problem.**

Table 3 has been removed.

Table 4 which is now Table 3 considers MOS-HIV scores by hypertension status which meets the minimum suggested by the reviewer.

**Table 4: the analysis here should be a logistic regression model with case (hypertension) as the outcome and quality of life as the exposure. That’s how to analyze case control studies.**

Thank you for your comment. The study is not a case control and thus we have made its cross-sectional nature. Hypertension is being considered as a main exposure and not the main outcome.

**Minor comments**

**Don’t put unnecessary zeroes in front of numbers, e.g. 07. Just write 7.**

**Tables 4 and 5 are too squashed and hard to read. Use landscape orientation when you need more width.**

Thank you for your comment. These have been revised.

**Ordinal variables should be displayed in their order. Look at education in Table 1. It makes no sense to display it that way. The order should be ‘no formal – primary – secondary – other tertiary – university’, so that any trend relationship will be visible.**

**Responses:** Thank you for your comment. The variables have been updated accordingly.

Reviewer #2: This is an interesting study on health-related quality of life in people living with HIV, stratified by the presence of hypertension. The study population comprises patients who have on average spent more than 15 years on ART and had their quality of life measured by accepted scores. I find the study very topical in terms of long-term co-morbidities for people living with HIV. My comments are as follows:

**The authors write: “Our findings suggest that hypertension reduces the quality of life” and “Presence of hypertension lowers the HRQoL of PWH on ART”. What came first: the egg or the hen? Could it also been those medical conditions, such as obesity (among others), lower the quality of life (mobility!), but also affect hypertension: if yes, hypertension would be on the causal pathway from those conditions on the outcome, acting as mediator. Maybe both is true. I suggest devoting a detailed and thorough paragraph on this in the discussion and reflect those discussions in the conclusions.**

Thank you for your comment. The discussion and conclusion have been revised accordingly to incorporate this comment.

**At the very end of the manuscript unmeasured confounders are mentioned, such as obesity and lifestyle factors. To what degree, and in which direction, may this influence the results?**

Thank you for your comment. These were included as areas for future research since they were not assessed in our study, line 310- 312.

**Definition of hypertension: I understand the definition to mean “history of hypertension”. This should be reflected in all interpretations: i.e. `history of’ rather than current hypertension.**

**Responses:** Thank you for your comment. Our definition for current hypertension is because those we selected were taking antihypertensives. We have added more description to clarify that those who were previously diagnosed and were currently on antihypertensive drugs were considered.

**While very basic summaries are given, I find it difficult to understand what range the quality-of-life scores have and what relevant differences would be.**

Thank you for your comment. The quality-of-life scores ranged between 0-100s

**I would add 2-3 sentences to the descriptive results and indicate somewhere what differences may be regarded as relevant.**

Thank you for your comment.

**Table 1: please delete p-values. They are of no use here, in a descriptive Table 1.**

Thank you for your comment. This has been implemented.

**Related: I would avoid unnecessary dichotomizations and speak of “insignificance”: Firstly, we can never ``prove'' a null hypothesis. Second, it is better to think in gradations of evidence rather than relying on binary cutoffs, see Rafi and Greenland for a great discussion (1).**

Thank you for your comment. The reference is very insightful regarding the use of P values and significance. Thank you for the comment. The age has been rerun as continuous variable.

**I find the issue of binarization particularly relevant for the linear model, Table 4:**

**i) Do not distinguish between significant/insignificant based on alpha=0.05. ii) interpret the coefficients in light of the above-mentioned relevance in differences in the health-related quality of life score: what do the estimates exactly mean? Give an exact interpretation in the results.**

Thank you for your comment. The distinguishing between significant and non-significance has been removed. The interpretation of the results is now added.

**iii) the multivariable model: it is unclear how variables were in-/excluded.**

Thank you for your comment. This has been included in the description for the methods.

Model selection uncertainty may be a relevant issue and I strongly advise to (additionally) report the full model, without model selection, including confidence intervals, context-specific parameter interpretation, and omitting of p-values.

iv) please provide a plot regarding the distribution of the residuals (i.e. approximate normality of residuals), and a plot on the home-/heteroscedasticity assumption.

Thank you for your comment.

**What I like about the data is that income has been captured, which may be a proxy for socio-economic status. This is a strength that can be mentioned in the discussion. I would personally also list the results in Ugandan Shillings, as exchange rates change.**

Thank you for your comment. The income has been changed to Uganda shillings. See table 1.

**- Table 3: the cutoffs do not make sense to me: first, with the 100.000 cutoff, there is hardly anyone in the third group, again making the p-values uninformative. Second, why sticking to the 50 copies cutoff? A detected viral load <1000 copies may still be regarded as a reasonably suppressed patient. If I had to pick, I would just do 2 groups.**

Previous Table 3 has been deleted.

**There are 5 tables, which is a lot. Table 4 could also be presented graphically. But this is just an option.**

Thank you for your comment. Previous Table 3 has been deleted.

**Why do some tables list mean +/- SE instead of the confidence interval?**

Thank you for your comment. The standard deviation is a measure of the spread of the data. However, we agree that the confidence intervals may be more informative.

1. Rafi Z, Greenland S. Semantic and cognitive tools to aid statistical science: replace confidence and significance by compatibility and surprise. BMC Med Res Methodol. 2020;20(1):244.
