# Supplementary material for: Health-related quality of life and hypertension in people with HIV on long-term antiretroviral therapy in Uganda
Source: PLoS One. 2024 Aug 8;19(8):e0306928. doi: 10.1371/journal.pone.0306928 (PMC11309503; doi:10.1371/journal.pone.0306928)
Supplement: Attachment — Submitted filename: Response to reviewers comments.docx [file pone.0306928.s002.docx]

**Thank you for the comments. These have been helpful in improving the manuscript.**

**Response to the Comments**

1. In the abstract the utility score phrase is repeated.

*This has been revised. We have removed the repeated phrase.*

2. Please ensure the access to the data are working (Figshare: <http://10.0.23.196/m9.figshare.20014799>)

*This has been updated and the access to the raw data is now open to the public.*

3. Clarify the inclusion criteria for HTN patients> in Line 105 it is said to be at least 3 records >140/90 but in line 115 it is said to be diagnosed people, receiving HTN treatment or >140/90. In addition, the rational for selecting only people +10 years on ART is unclear and not explained in the text.

*This has been revised and updated line 106 to 112. It reads as follow: We recruited PWH with or without hypertension who had been on ART for at least 10 years and were in the ALT cohort. The prolonged exposure to certain classes of ART (≥ 10 years) and HIV could increase their risk of developing hypertension hence their selection. Participants with hypertension were entered into this study based on the existing ALT Cohort standard operating procedure. The ALT Cohort standard operating procedures defined hypertension as three systolic measurements above 140 mm Hg or diastolic measurement above 90 mm Hg or history of treatment with antihypertensive drugs (as per ICEA) or patient-reported hypertension*

4. Line 118 seems incomplete

*This has been revised.*

5. Line 127 seems a typo in " EQ5D-5Lquestionnaire"

*This has been revised.*

6. Line 132: It is suggested to change 'assuming' to 'assuming an equal distribution of participants in each group' for greater clarity."

*This has been revised.*

7. Line 150: In the following text, there is an extra dot after '2020': 'At the time of the study (mid-year 2020.) and analysis...'"

*This has been revised.*

8. Line 163: I think there is a discrepancy in the name of the questionnaire in the text. It reads E5Q-5D-5L, but I believe it should be EQ-5D-5L.

*This has been corrected.*

9. Line 189: The word "hypertension" should be in lowercase.

*This has been corrected.*

10. Line 198:"In the word 'differences', there is an extra space."diffe-rences"

*This has been corrected.*

11. Line 219-220: The wording can be improved as follows "More participants without hypertension were in stage IV of the WHO HIV staging compared to those with hypertension (41.8% vs 35.1%, respectively)."

*This has been revised and corrected, line 217-218.*

**Discussion**

12. Line 290 propose those who disclosed their status had lower QoL because discrimination, but more data is needed for this, as those who do not disclose could have higher internal stigma, other potential causes should be identified for this situation.

*It is true that those who did not disclose their HIV status could experience internalised stigma which could ultimately affect their HRQoL, and this is a valid concern. We believe that this is a complex question that cannot be addressed in the present study and would require a robust research study to understand the dynamics involved between disclosure, stigma and health outcomes in PWH. This has been stated a future research area for the scientific community to explore, line 385 and 386.*

13. Do patients with HTN have integrated care? or they need to go more frequently to the hospital or laboratory services? How many pills they had to take, are the HTN pills provided for free? Those factors could impair QoL and were not mentioned.

*We recognise that pill burden, cost of pills as well as inconveniences in accessing care could impart HRQoL. However, this was not explored in this study and has been included as a limitation.*

14. COVID19 was estimated to affect QoL in people with higher income, as was at this time the study happened? This suggest that authors should provide a clearer contextual information about how COVID19 could have impacted all the other dimensions of QoL among people with or without HTN, it is that HTN services were closed? Etc

*This has been revised and updated illustrating how the restriction imposed during the COVID-19 could have affect the HRQoL of PWH with or with hypertension, line 320-323.*

**Limitations**

15. The authors should state important limitations
    a) No validated EuroQol for Uganda
    b) Significant age difference with the comparator
    c) Population no representative of all HIV (+10 year retained in services)
    b) No data on obesity, diabetes, HTN treatment and pocket costs that could have impacted results.

*The limitations have been revised to reflect this, line 329- 342.*

**Reviewer #1:** I don't have any comments. I think the call is very pertinent the calls for inclusion of quality-of-life assessment in the management of PWH who have been diagnosed with hypertension to identify those at risk and plan early interventions.

***Thank you for the comment.***

**Reviewer #2:** In this study, Charles Batte and his colleagues explore the impact on the quality of life of people living with HIV and hypertension. Overall, I consider it a valuable work that provides important information. However, I believe it would be beneficial to include additional information about the antiretroviral regimens received by the participants. This is a relevant topic, especially because it has been suggested that integrase inhibitors (INSTIs) may be associated with a potential increase in blood pressure. Additionally, it would be pertinent to discuss the possible impact on the quality of life of these regimens due to factors such as weight gain and the risk of developing new conditions such as diabetes and/or hypertension. I suggest reviewing the provided link to access additional information, as I was unable to view the data when attempting to access it directly through the browser or by accessing figshare: <http://10.0.23.196/m9.figshare.20014799>.

The rest of my observations are minor.
